# H_3_O^+^ tetrahedron induction in large negative linear compressibility

**DOI:** 10.1038/srep26015

**Published:** 2016-05-17

**Authors:** Hui Wang, Min Feng, Yu-Fang Wang, Zhi-Yuan Gu

**Affiliations:** 1 School of Physics and Engineering, Henan University of Science and Technology, Luoyang 471003, China; 2 Department of Physics, Nankai University, Tianjin 300071, China; 3 College of Chemistry and Materials Science, Nanjing Normal University, Nanjing 210023, China

## Abstract

Despite the rarity, large negative linear compressibility (NLC) was observed in metal-organic framework material Zn(HO_3_PC_4_H_8_PO_3_H)∙2H_2_O (ZAG-4) in experiment. We find a unique NLC mechanism in ZAG-4 based on first-principle calculations. The key component to realize its large NLC is the deformation of H_3_O^+^ tetrahedron. With pressure increase, the oxygen apex approaches and then is inserted into the tetrahedron base (hydrogen triangle). The tetrahedron base subsequently expands, which results in the *b* axis expansion. After that, the oxygen apex penetrates the tetrahedron base and the *b* axis contracts. The negative and positive linear compressibility is well reproduced by the hexagonal model and ZAG-4 is the first MOFs evolving from non re-entrant to re-entrant hexagon framework with pressure increase. This gives a new approach to explore and design NLC materials.

Most materials contract in all directions when hydrostatically compressed. That is the volume (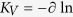

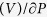
), area (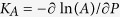), and linear (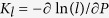) compressibility are all positive^1^. Negative volume compressibility is thermodynamically impossible^2^. Counterintuitively, negative linear compressibility (NLC) indeed occurs in rare and remarkable crystals^1^,^3^,^4^,^5^,^6^. After screening of reported elastic constant tensors from approximately five hundred crystals, Baughman *et al*. uncovered only 13 compounds showing negative compressibility in simple inorganic or organic compounds^1^. Among the thirteen crystals, 11 structures were of monoclinic or lower symmetry. The typical positive linear compressibility (PLC) for crystal material lies in the range *K*_*l*_ ≈ 5–20 TPa^−1^, with lattice parameter contracting 0.5~2% for each GPa increase in pressure^7^. Unfortunately, experimentally determined NLC, for a long time, had been below −2 TPa^−1^ (−0.2 TPa^−1^ for LaNbO_4_^8^, −1.2 TPa^−1^ for Se^9^ and −2 TPa^−1^ for BAsO_4_^10^). Until recently, stronger NLC behavior is found: −3.8 TPa^−1^ for methanol monohydrate from 0 to 0.5 GPa at 160K^11^, −6.4 TPa^−1^ for α-BiB_3_O_6_ from 0 to 6.5 GPa^12^, −12 TPa^−1^ for KMn[Ag(CN)_2_]_3_ from 0 to 2.2 GPa^13^, −41 TPa^−1^ for [Fe(dpp)_2_(NCS)_2_]∙py from 0 to 0.5 GPa^14^, −42 TPa^−1^ for Zn[Au(CN)_2_]_2_ from 0 to 1.8 GPa^15^ and −75 TPa^−1^ for Ag_3_[Co(CN)_6_] from 0 to 0.19 GPa^7^,^16^. Contrary to conventional materials, a specific direction of NLC material could not only increase with the increase of hydrostatic pressure, but also remain invariant^15^. Therefore, NLC is a highly desirable property exploitable in the development of artificial muscles^17^, extremely sensitive pressure detectors, shock resistance materials and *etc*.

Metal-organic frameworks (MOFs) with extreme surface area and tunable pore structure have revolutionized the field of crystal engineering^18^,^19^,^20^,^21^. They consist of metal ions and organic linkers, exhibiting various unique physical and chemical properties for diverse applications^22^,^23^,^24^. The crystalline order between metals and ligands combined with cooperative structural transformability, forming flexible and responsive MOFs, namely soft porous frameworks. These materials can respond to mechanical stimuli in a tunable and precise fashion by molecular design, which does not exist for other known solid-state materials^25^,^26^. The elastic behaviour of soft porous crystals is usually complex, such as anisotropic flexibility, negative Poisson’s ration and high NLC^25^,^26^,^27^. The investigation of MOFs structure deformation under pressure can not only reveal the mechanism of these behaviours, but also can help us design new MOFs with desired mechanical property. The research of the negative linear/area compressibility of framework materials started very recently^28^,^29^,^30^,^31^. The first case of NLC in MOFs was [NH_4_][Zn(HCOO)_3_], which showed a high degree of mechanical anisotropy and negative compressibility *K*_*l*_ = −1.8 TPa^−1^ along its *c* axis from 0 to 0.94 GPa^32^. After that, NLC was found in silver(I) 2-methylimidazolate with *K*_*l*_ = −4.3 TPa^−1^ (along *c* axis, from 0 to 1 GPa)^33^ and [Ag(en)]NO_3_-I with *K*_*l*_ = −28.4 TPa^−1^ (along *a* axis, from 0 to 0.92 GPa)^34^. Clearfield and others pioneered named the MOFs formed from the linker molecules with alkyl chains as zinc alkyl gate(ZAG) because of the likeness of the structure to a child safety gate (Fig. 1(a))^21^,^35^. Recently, Gagnon *et al*. measured the lattice parameters of ZAG-4 under pressure with single crystal X-ray diffraction and observed NLC^36^,^37^. The *b* axis of ZAG-4 increases almost 2% in the range of 1.65–2.81 GPa, indicating a strong NLC (*K*_*l*_ ≈ −16 TPa^−1^). Due to the inherently small atomic scattering factor of hydrogen, the exact positions of H_2_O in ZAG-4 can not be easily detected by X-ray diffraction technique^38^. Aurelie U Ortiz *et al*. calculated the ZAG-4 structure under pressure, found a proton transfer and attributed the NLC (from 1.65 to 2.81 GPa) to this structural transition^39^. However, PLC was observed after NLC in experiment. This explanation did not answer why NLC and PLC occurs subsequently after proton transfer. Therefore, an unambiguous mechanism for the NLC in ZAG-4 is still an unresolved matter.

Density functional calculation, an integral part of MOFs research, is complementary to experimental techniques and offers invaluable information in characterization and understanding of systems^27^,^40^,^41^,^42^. In order to elucidate the NLC mechanism of ZAG-4, we performed the density functional calculation using both PBE and Wu-Cohen (WC) functional^43^ as implemented in the Quantum Espresso package^44^ to determine their atomic structures under pressure. The PBE functional, usually overestimating lattice parameters, has been widely used in density functional calculations and the WC functional is known to be accurate in predicting solid volumes^45^,^46^,^47^. The wave function was expanded in a plane-wave basis set with an energy cutoff of 70 Ry and the first Brillouin zone was sampled on a 3 × 3 × 4 mesh. The ultra-soft pseudopotential was used to represent the electron-ion interaction.

The conventional unit cell of ZAG-4 is depicted in Fig. 1^21^,^35^,^36^. It has a base-centered monoclinic lattice with the *b* axis perpendicular to the *a*-*c* plane. Herein, the three building blocks of ZAG-4 are the inorganic Zn-O-P-O chains and two bridging ligands (C_4_H_8_ and H_2_O). The 1D Zn-O-P-O chains orient along the *c* axis and are linked with each other along the *b* axis by H_2_O molecules. So as to give a detailed description of the linkage between H_2_O molecules and Zn-O-P-O chains, we enlarge the bridging zone in Fig. 1(c). The ZnO_4_ and PO_3_C tetrahedrons are linked along the *c* axis by sharing oxygen atoms and form the Zn-O-P-O chains. The water molecules are located between two Zn-O-P-O chains. As a result, Zn-O-P-O chains bridged by H_2_O form an inorganic 2D structure parallel to the *b*-*c* plane. Furthermore, the inorganic planes are linked with each other along the *a* axis by C_4_H_8_, which is directly bonded with the P cations. In brief, the 3D framework is established with the inorganic Zn-O-P-O chains extending along the *c* axis linked with each other by C_4_H_8_ chains and H_2_O molecules along the *a* and *b* axis, respectively.

Firstly, we use the experimental crystal structure at zero pressure as our initial point and fully relax the lattice parameters and atomic positions with PBE functional. The calculated lattice parameters (*a* = 19.00 Å, *b* = 8.41 Å, *c* = 8.18 Å, and volume = 1214.27 Å^3^) agree well with experimental results (*a* = 18.51 Å, *b* = 8.29 Å, *c* = 8.27 Å, and volume = 1160.55 Å^3^). As the calculated volume is slight larger than the experimental value, our calculated bulk modulus of (11.6 GPa) is slightly lower than the experimental result (11.7 GPa). Although it is about one thirty-fifth of the bulk modulus of *sp*^3^ carbon allotrope (around 400 GPa)^48^,^49^, this value is higher than that of porous MOFs MIL-53 and NH_2_-MIL-53^30^,^50^, but is lower than that of dense MOFs^51^,^52^,^53^,^54^.

In order to explore its NLC mechanism, we applied hydrostatic pressure to ZAG-4 and investigate its structure variation. The calculated lattice parameters, accompanied with available experimental data^36^, are shown in Fig. 2. We increase pressure and take the previously optimized structure as the initial point of higher pressure condition. In this way, we increase pressure to 8 GPa and optimize the structure step by step.

As can be seen in Fig. 2, the experimental lattice parameters are well reproduced by our calculation based on the PBE functional. It is well-known that PBE calculation typically overestimates the lattice parameters by 1–2%. As far as the overestimation is concerned, our calculated results agree excellently with the experimental data. As pointed by Gagnon *et al*., the alkyl chains serve like a spring cushion and hence contract much under pressure^36^. With pressure increasing from zero to 2 GPa, the calculated volume compressibility is around 71 TPa^−1^, and that of the experimental value from zero to 1.65 GPa is around 69 TPa^−1^. The calculated *b* and *c* axis have a jump from 2.25 to 2.5 GPa, which is accompanied with the proton transfer. Below 2.25 GPa, the H_1_ is close to the PCO_3_ octahedron and far away from the H_2_O (Fig. 1(c)). When pressure increases to 2.5 GPa, the proton turns to be close to the H_2_O and forms the H_3_O^+^ tetrahedron. Aurélie U Ortiz *et al*. also found this proton transfer and the H_3_O^+^ tetrahedron formation^36^,^39^. They used the wine-rack motif to explain the NLC of ZAG-4 after the proton transition. However, as show in Fig. 2b, NLC does not occur immediately after proton transfer. Instead, the *b* axis smoothly expands from 5 to 6.25 GPa (blue shaded area in Fig. 2(b)), which is far away from the proton transfer pressure (2.5 GPa). This indicates that proton transfer is not enough to lead to the NLC of ZAG-4. There must be something new.

We repeat these calculations with the WC functional, because the lattice constants of solids as determined by it are between LDA and PBE results and on average closer to experiment^45^,^46^,^47^. In the WC results of ZAG-4, the H_3_O^+^ tetrahedron is formed at zero pressure. Consequently, the jump from 2.25 to 2.5 GPa in Fig. 2 does not exist in WC functional results (Supplementary Information). Although the H_3_O^+^ is formed at zero pressure, the NLC does not take place immediately from zero pressure. Instead, the NLC of the *b* axis occurs in WC functional results from 3.5 to 4.75 GPa. This means the H_3_O^+^ tetrahedron and the NLC of *b* axis are reproduced in WC functional results as well.

We now pay our attention to the changes under pressure of the *b* axis. The experimentally observed NLC is from 1.65 to 2.81 GPa with the *b* axis increasing 1.8% (red shaded area in Fig. 2(b))^36^. The experimental data are too few (only two) to give a detailed description of the lattice parameters evolution. So as to draw a complete picture, we calculate the lattice parameters from 5 to 7 GPa with a small pressure step of 0.25 GPa. As shown in Fig. 3(a), the *b* axis increases from 5 GPa and reaches its maximum at 6.25 GPa. Accordingly, the average NLC from 5 to 6.25 GPa is −11 TPa^−1^, as strong as that of KMn[Ag(CN)_2_]_3_ (−12 TPa^−1^, from 0 to 2.2 GPa)^13^. After that, the *b* axis decreases with pressure increase, and hence the linear compressibility turns to be positive.

It is the deformation of the H_3_O^+^ tetrahedron that leads to the NLC of the *b* axis. As shown in Fig. 1(d,e), the apex oxygen of the H_3_O^+^ tetrahedron is above the H triangle at 5 GPa. The distance (*d*_O-H triangle_) between the apex oxygen and the H triangle is defined to be positive at this condition (left inset of Fig. 3(a)). Comparing the left and right inset of Fig. 3(a) (or Fig. 1(e,f)), we find that *d*_O-H triangle_ turns from positive to negative with pressure increase. At the same time, evolution of *d*_O-H triangle_ is completely accompanied with the evolution of the *b* axis from expansion to contraction. We also calculate the structure of dehydrated ZAG-4. By deleting the H_2_O molecules in ZAG-4 at zero pressure, we get the initial structure of dehydrated ZAG-4. Following the process of Fig. 2, we increase pressure and optimize the structure step by step. The calculated results (Supplementary Information) show that the *b* axis of dehydrated ZAG-4 decreases smoothly with pressure increase. This means that the H_3_O^+^ tetrahedron deformation is essential to the NLC of ZAG-4.

It is easy to understand the NLC mechanism of ZAG-4 with the following picture in mind. Initially, *d*_O-H triangle_ of the H_3_O^+^ tetrahedron is positive at low pressure. The apex oxygen moves towards the H triangle with pressure increase. It leads to the decrease of *d*_O-H triangle_ (Fig. 3(a)), the area expansion of hydrogen triangle (Fig. 3(b)) and the expansion of the *b* axis (Fig. 3(a)). Therefore, the NLC of the *b* axis is directly results from the H_3_O^+^ tetrahedron deformation. With further pressure increase, the apex oxygen penetrates the H triangle and *d*_O-H triangle_ turns to be negative (right inset of Fig. 3(a)). From then on, the apex oxygen moves away from the H triangle. Consequently, both the area of the H triangle and the *b* axis decrease with pressure increase. The calculated results using Wu-Cohen functional also show these characteristic features of H_3_O^+^ tetrahedron deformation. In brief, the *b* axis is expanded by the apex oxygen approaching the H triangle, and is contracted by the apex oxygen moving away from the H triangle.

There are four microscopic mechanisms frequently used: (i) ferroelastic phase transition, (ii) polyhedral tilt, (iii) helical system, and (iv) wine-rack, honeycomb or related topology.^6^ The NLC and PLC mechanism of ZAG-4 can be explained by the hexagonal model^55^,^56^. The non re-entrant and re-entrant hexagons are illustrated in the left and right inset of Fig. 4, respectively. The linear compressibility *K*_*l*_ of this mode can be given analytically as^56^


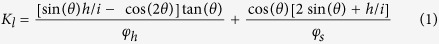


in which, as illustrated in the left inset of Fig. 4, *i* and *h* are the lengths of the inclined and horizontal ribs, respectively; *θ* is the angle between the inclined rib and the vertical direction; *φ*_h_ and *φ*_s_ are the hinging and stretching force constants per unit thickness of the hexagonal plane, respectively. The first term of equation (1) assumes that ribs are rigid along their lengths and the deflection totally originates from the change of angle *θ*, while the second one represents that hexagon deforms solely through stretching of ribs and *θ* does not change at all.

With pressure increase, *θ* deceases from positive to negative, which is similar to the H_3_O^+^ tetrahedron deformation depicted in the inset of Fig. 3(a). Figure 4 shows the calculated linear compressibility with *i*/*h* = 3 and *φ*_s_/*φ*_h_ = 10. Similarly with the *b* axis evolution in Fig. 3(a), the left side of Fig. 4 is NLC (34° > *θ* > 3°) and the right side is PLC (*θ* < 3°). There are two H_3_O^+^ tetrahedrons with positive *d*_O-H triangle_ in Fig. 1(d). This condition is similar to the non re-entrant hexagon with NLC. When *d*_O-H triangle_ becomes negative, it turns to be similar to the re-entrant hexagon with positive NL. Therefore, the NLC mechanism of ZAG-4 can be simplified to the hexagonal model.

In conclusion, based on the first-principle calculations, we investigated the crystal structure of ZAG-4 under pressure. Our calculated evolutions of the lattice parameters with pressure excellently agree with the experimental results. The NLC of ZAG-4 occurs in the pressure span of 1.5 GPa (1.16 GPa) with the *b* axis increasing 1.2% (1.8%) in calculation (experiment). By inspecting the evolution of atomic position with pressure, we found that the H_3_O^+^ tetrahedron deformation is the key point of understanding the large NLC. Initially, with pressure increase, the apex oxygen moves toward the tetrahedron base and expands the H triangle and the *b* axis. After penetrating the H triangle, the apex oxygen moves away and then the *b* axis contracts with pressure increase. The NLC characteristic of ZAG-4 is well reproduced by the hexagonal model, which gives a vivid explanation for the linear compressibility switch from negative to positive values. Usually, crystal has either non re-entrant or re-entrant hexagon framework. ZAG-4 is the first MOFs evolving from non re-entrant to re-entrant hexagon framework with pressure increase. Our finding prescribes a general way to obtain the rare NLC property in complex structures. As H_2_O molecules abundantly exist in inorganic and organic materials, it will prompt more investigations on negative compressibility in complex materials^57^.

## Additional Information

**How to cite this article**: Wang, H. *et al*. H3O^+^ tetrahedron induction in large negative linear compressibility. *Sci. Rep.* **6**, 26015; doi: 10.1038/srep26015 (2016).

## Supplementary Material

Supplementary Information

## Figures and Tables

**Figure 1:**
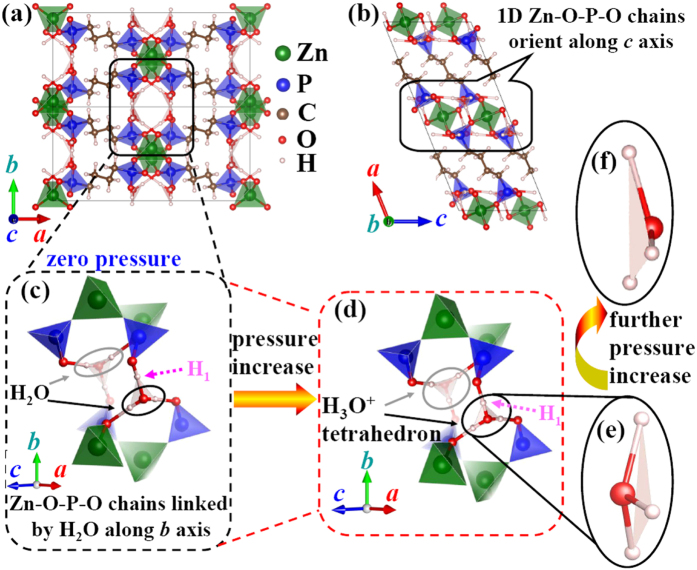
Structural details of ZAG-4. (**a**) ZAG-4 viewed along [0 0 1] direction. (**b**) ZAG-4 viewed along [0 1 0] direction. (**c**) Partial enlargement of (**a**), viewed approximately along [1 0 1] direction. At zero pressure, H_1_ is close to PCO_3_ and far away from H_2_O. (**d**) H_1_ moves away from PCO_3_ with the increase of pressure, and then H_3_O^+^ tetrahedron is formed. (**e**) Side view enlargement of H_3_O^+^ tetrahedron. (**f**) With further pressure increase, apex oxygen penetrates H_3_O^+^ tetrahedron base and moves to the other side of hydrogen plane.

**Figure 2:**
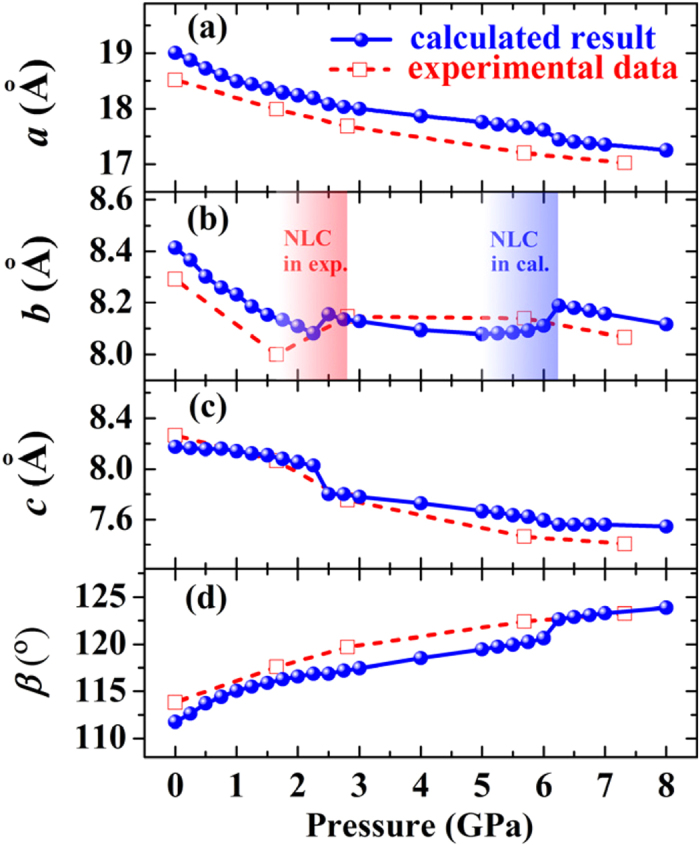
Experimental and calculated lattice parameters. Red and blue shaded areas in (**b**) manifest the NLC zone in experiment (from 1.65 to 2.81 GPa with increase of 1.8% in *b* axis) and calculation (from 5 to 6.25 GPa with the increment of 1.4% in *b* axis), respectively.

**Figure 3:**
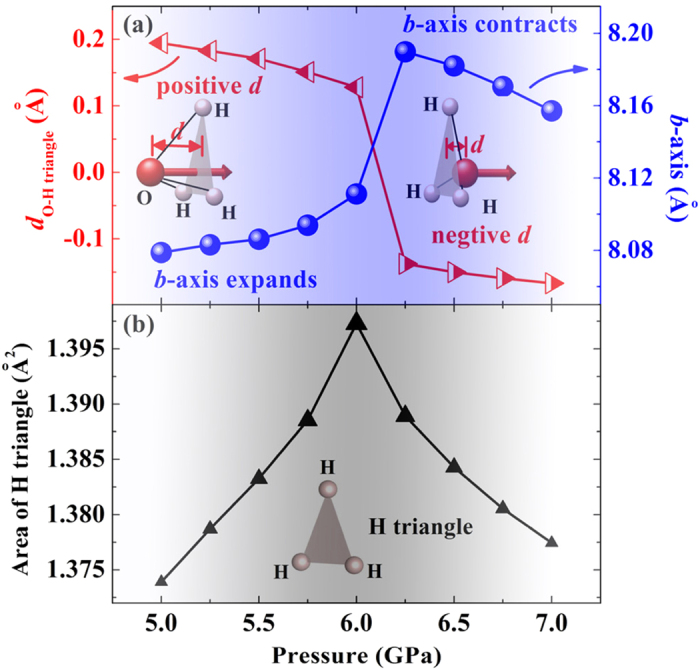
(**a**) Evolutions of the distance (*d*_O-H triangle_) between apex oxygen and H triangle and the *b* axis. (**b**) Area of the H triangle. With pressure increase, the apex oxygen approaches the H triangle and expands its area and the *b* axis. After the apex oxygen penetrates the H triangle, the area of the H triangle decreases and the *b* axis contracts.

**Figure 4:**
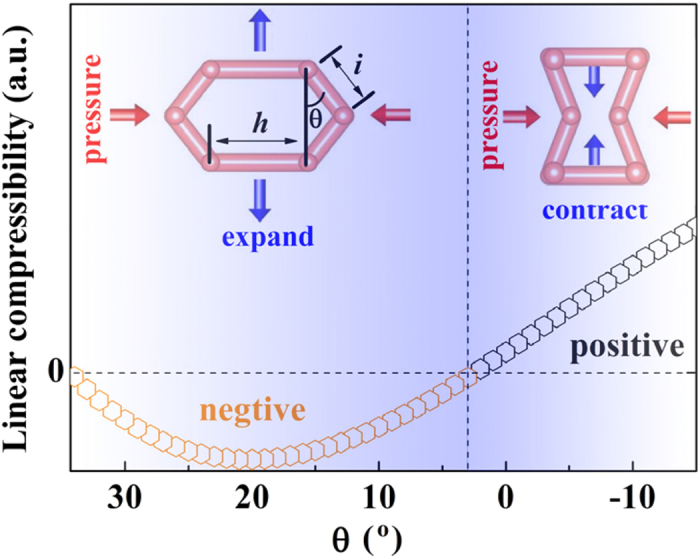
Linear compressibility of hexagonal model. The left inset is a non re-entrant hexagon with *θ* > 0, while the right one is a re-entrant hexagon with *θ* < 0. The hexagons and crystal structures were drawn using the VESTA software^57^.
