# Supplementary material for: H3O+ tetrahedron induction in large negative linear compressibility
Source: Sci Rep. 2016 May 17;6:26015. doi: 10.1038/srep26015 (PMC4868991; doi:10.1038/srep26015)
Supplement: Supplementary Information [file srep26015-s1.doc]

**Supplementary Information**

**H_3_O^+^ tetrahedron induction in large negative linear compressibility**

Hui Wang*, Min Feng, Yu-Fang Wang, Zhi-Yuan Gu*

**Figure S1.** Experimental and calculated lattice parameters with WC functional.

**Figure S2.** Simulated XRD patterns of ZAG-4.

**Figure S3.** Calculated lattice parameters of dehydrated ZAG-4.


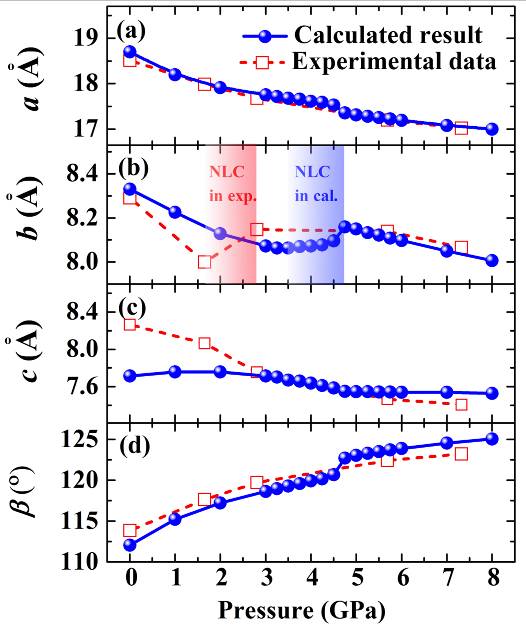


Figure S1. Experimental and calculated lattice parameters with WC functional. Red and blue shaded areas in (b) manifest the NLC zone in experiment (from 1.65 to 2.81 GPa) and calculation (from 3.5 to 4.75 GPa), respectively.


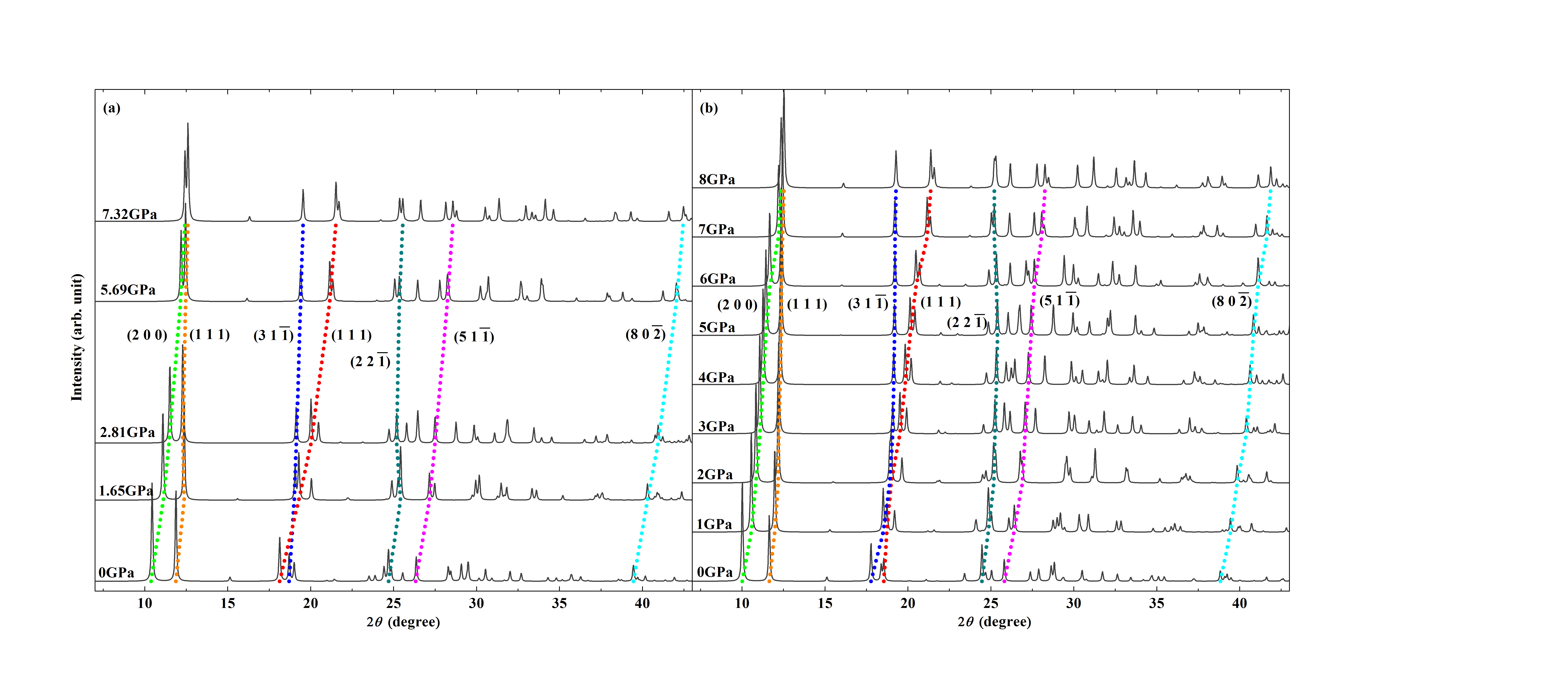


Figure S2. Simulated XRD patterns of ZAG-4 with λ=1.54Å. (a) The XRD patterns are calculated based on experimentally obtained crystal structures^1^. (b) The XRD patterns are calculated based on our calculated crystal structures. The dotted lines are guides to the eye.

1. Gagnon, K.J., Beavers, C.M. & Clearfield, A. *J. Am. Chem. Soc.* **135**, 1252-5 (2013).


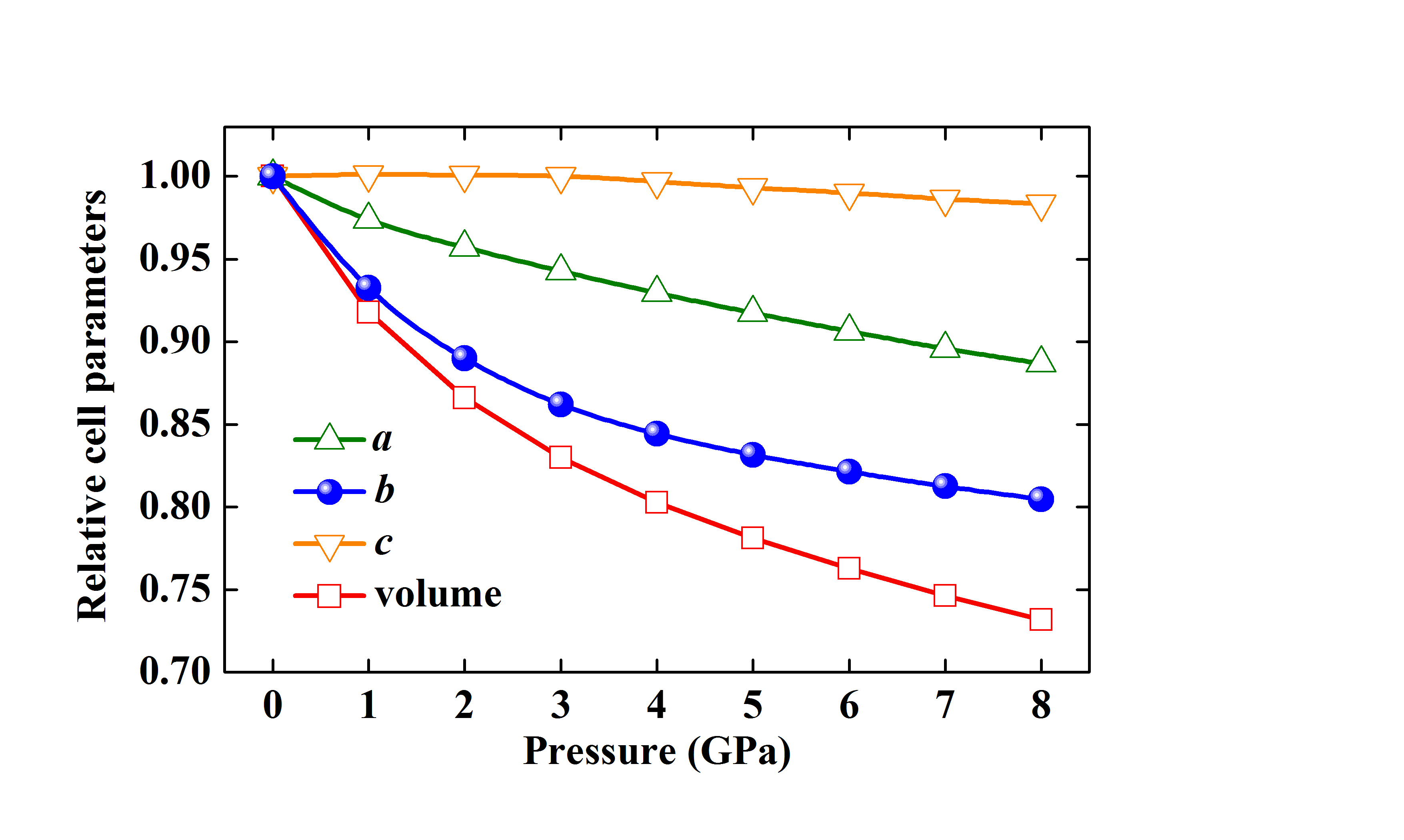


**Figure S3.** Calculated lattice parameters of dehydrated ZAG-4 with PBE functional. The *b* axis decreases smoothly with pressure increase. The lattice parameters of conventional unit cell at zero pressure: *a*=21.20Å, *b*=5.96Å, *c*=9.23Å and volume= 992.29 Å3.
